# Bis(dipyridin-2-yl­amine-κ^2^ *N* ^2^,*N* ^2′^)palladium(II) dinitrate

**DOI:** 10.1107/S1600536812012081

**Published:** 2012-03-31

**Authors:** Kwang Ha

**Affiliations:** a School of Applied Chemical Engineering, The Research Institute of Catalysis, Chonnam National University, Gwangju 500-757, Republic of Korea

## Abstract

The asymmetric unit of the title compound, [Pd(C_10_H_9_N_3_)_2_](NO_3_)_2_, contains one half of a cationic Pd^II^ complex and one NO_3_^−^ anion. In the complex, the Pd^II^ ion is four-coordinated by four pyridine N atoms derived from the two chelating dipyridin-2-yl­amine (dpa) ligands. The Pd^II^ atom is located on an inversion centre, and thus the PdN_4_ unit is exactly planar. The dpa ligand itself is not planar, showing a dihedral angle between the pyridine rings of 39.9 (1)°. The anions are connected to the complex by inter­molecular N—H⋯O hydrogen bonds between the two O atoms of the anion and the N—H group of the cation. Weak inter­molecular C—H⋯O hydrogen bonds additionally link the constituents in the crystal structure. The NO_3_^−^ anion was found to be disordered over two sites with a site-occupancy factor of 0.55 (10) for the major component.

## Related literature

For the crystal structures of the related cationic Pd^II^ complexes [Pd(dpa)_2_](*X*)_2_ (*X* = Cl or PF_6_), see: Živković *et al.* (2007 ▶); Antonioli *et al.* (2008 ▶).
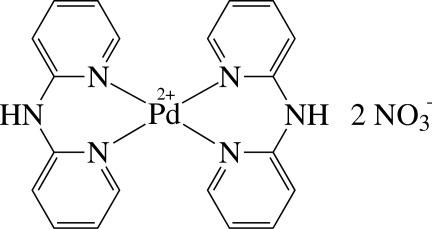


## Experimental

### 

#### Crystal data

[Pd(C_10_H_9_N_3_)_2_](NO_3_)_2_
*M* *_r_* = 572.82
Monoclinic, 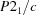

*a* = 8.5760 (8) Å
*b* = 16.8916 (16) Å
*c* = 7.4893 (7) Å
β = 96.296 (2)°
*V* = 1078.37 (18) Å^3^
*Z* = 2
Mo *K*α radiation
μ = 0.92 mm^−1^
*T* = 200 K
0.29 × 0.23 × 0.14 mm

#### Data collection

Bruker SMART 1000 CCD diffractometer
Absorption correction: multi-scan (*SADABS*; Bruker, 2000 ▶) *T*_min_ = 0.890, *T*_max_ = 1.000
7678 measured reflections
2626 independent reflections
1932 reflections with *I* > 2σ(*I*)
*R*_int_ = 0.025

#### Refinement

*R*[*F* ^2^ > 2σ(*F* ^2^)] = 0.032
*wR*(*F* ^2^) = 0.086
*S* = 1.16
2626 reflections
170 parameters
H-atom parameters constrained
Δρ_max_ = 0.91 e Å^−3^
Δρ_min_ = −0.94 e Å^−3^

### 

Data collection: *SMART* (Bruker, 2000 ▶); cell refinement: *SAINT* (Bruker, 2000 ▶); data reduction: *SAINT*; program(s) used to solve structure: *SHELXS97* (Sheldrick, 2008 ▶); program(s) used to refine structure: *SHELXL97* (Sheldrick, 2008 ▶); molecular graphics: *ORTEP-3* (Farrugia, 1997 ▶) and *PLATON* (Spek, 2009 ▶); software used to prepare material for publication: *SHELXL97*.

## Supplementary Material

Crystal structure: contains datablock(s) global. DOI: 10.1107/S1600536812012081/im2365sup1.cif

Additional supplementary materials:  crystallographic information; 3D view; checkCIF report

## Figures and Tables

**Table 1:** Hydrogen-bond geometry (Å, °)

*D*—H⋯*A*	*D*—H	H⋯*A*	*D*⋯*A*	*D*—H⋯*A*
N2—H2*N*⋯O2^i^	0.92	2.08	2.964 (4)	160
N2—H2*N*⋯O3*A*^i^	0.92	2.49	3.274 (17)	144
C2—H2⋯O1^ii^	0.95	2.57	3.409 (5)	148
C3—H3⋯O3*A*	0.95	2.55	3.23 (4)	129
C4—H4⋯O2^i^	0.95	2.37	3.152 (5)	140
C7—H7⋯O3*A*^i^	0.95	2.25	3.07 (2)	144
C10—H10⋯O2^iii^	0.95	2.53	3.334 (5)	142

Symmetry codes: (i) 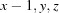; (ii) 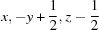; (iii) 
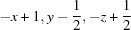
.

**Fig. 1.**
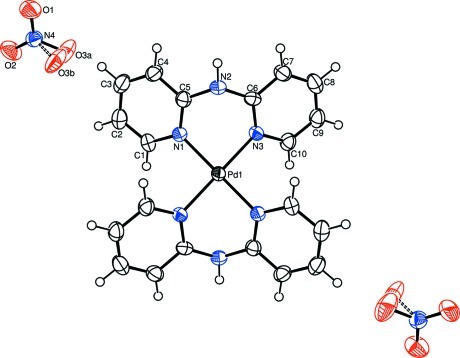
Molecular structure of the title compound, with displacement ellipsoids drawn at the 50% probability level for non-H atoms. Unlabelled atoms are related to the reference atoms by the (-x, -y, -z) symmetry transformation. The minor bond of the disordered anion is drawn as a dashed line.

**Fig. 2.**
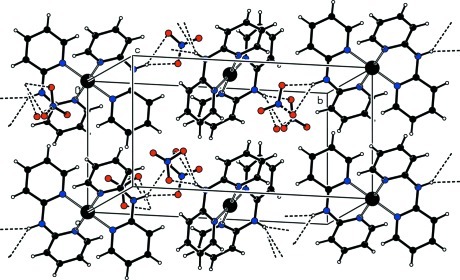
A view of the unit-cell contents of the title compound. Intermolecular N—H···O hydrogen-bond interactions are drawn as dashed lines.

## supplementary crystallographic information

### Comment

Crystal structures of related cationic Pd^II^ complexes, [Pd(dpa)_2_](*X*)_2_ (dpa = dipyridyl-2-ylamine, C_10_H_9_N_3_; *X* = Cl or PF_6_), have been reported previously (Živković *et al.*, 2007; Antonioli *et al.*, 2008).

The asymmetric unit of the title compound, [Pd(C_10_H_9_N_3_)_2_](NO_3_)_2_, contains one half of a cationic Pd^II^ complex and one NO_3_^-^ anion (Fig. 1). In the complex, the Pd^II^ ion is four-coordinated by four pyridine N atoms derived from the two chelating dipyridin-2-ylamine (dpa) ligands. The Pd atom is located on an inversion centre, and thus the PdN_4_ unit is exactly planar. The dpa ligand itself is not planar, showing a dihedral angle between the pyridine rings of 39.9 (1)°. The two Pd—N bond lengths are almost equivalent [Pd—N: 2.021 (3) and 2.030 (3) Å]. The anions are connected to the complex by intermolecular N—H···O hydrogen bonds between the two O atoms of the anion and the N—H group of the cation (Fig. 2 and Table 1). Weak intermolecular C—H···O hydrogen bonds additionally link the constituents in the crystal structure (Table 1). The complex molecules are stacked into columns along the *a* axis. In the columns, several intermolecular π-π interactions between the pyridine rings are present, the shortest ring centroid-centroid distance being 3.771 (2) Å.

### Experimental

To a solution of Pd(NO_3_)_2_.2H_2_O (0.1315 g, 0.494 mmol) in acetone (30 ml) was added dipyridin-2-pyridylamine (0.0858 g, 0.501 mmol) and stirred for 3 h at room temperature. The formed precipitate was separated by filtration and washed with ether, and dried under vacuum, to give a yellow powder (0.1110 g). Crystals suitable for X-ray analysis were obtained by slow evaporation from a CH_3_CN solution at room temperature.

### Refinement

Carbon-bound H atoms were positioned geometrically and allowed to ride on their respective parent atoms [C—H = 0.95 Å and *U*_iso_(H) = 1.2*U*_eq_(C)]. Nitrogen-bound H atom was located from Fourier difference maps then allowed to ride on its parent atom in the final cycles of refinement with N—H = 0.92 Å and *U*_iso_(H) = 1.5 *U*_eq_(N). The NO_3_^-^ anion displayed relatively large displacement factors and low electron density peaks so that the anion appears to be highly disordered. The atom O3 was modelled anisotropically as disordered over two sites with a site-occupancy factor of 0.55 (10) for the major component. The highest peak (0.91 e Å^-3^) and the deepest hole (-0.94 e Å^-3^) in the difference Fourier map are located 0.68 Å and 0.85 Å from the atoms N3 and Pd1, respectively.

### Crystal data

**Table:**

[Pd(C_10_H_9_N_3_)_2_](NO_3_)_2_	*F*(000) = 576
*M**_r_* = 572.82	*D*_x_ = 1.764 Mg m^−^^3^
Monoclinic, *P*2_1_/*c*	Mo *K*α radiation, λ = 0.71073 Å
Hall symbol: -P 2ybc	Cell parameters from 4149 reflections
*a* = 8.5760 (8) Å	θ = 2.4–28.3°
*b* = 16.8916 (16) Å	µ = 0.92 mm^−^^1^
*c* = 7.4893 (7) Å	*T* = 200 K
β = 96.296 (2)°	Block, yellow
*V* = 1078.37 (18) Å^3^	0.29 × 0.23 × 0.14 mm
*Z* = 2	

### Data collection

**Table:**

Bruker SMART 1000 CCD diffractometer	2626 independent reflections
Radiation source: fine-focus sealed tube	1932 reflections with *I* > 2σ(*I*)
Graphite monochromator	*R*_int_ = 0.025
φ and ω scans	θ_max_ = 28.3°, θ_min_ = 2.4°
Absorption correction: multi-scan (*SADABS*; Bruker, 2000)	*h* = −9→11
*T*_min_ = 0.890, *T*_max_ = 1.000	*k* = −22→20
7678 measured reflections	*l* = −9→4

### Refinement

**Table:**

Refinement on *F*^2^	Primary atom site location: structure-invariant direct methods
Least-squares matrix: full	Secondary atom site location: difference Fourier map
*R*[*F*^2^ > 2σ(*F*^2^)] = 0.032	Hydrogen site location: inferred from neighbouring sites
*wR*(*F*^2^) = 0.086	H-atom parameters constrained
*S* = 1.16	*w* = 1/[σ^2^(*F*_o_^2^) + (0.011*P*)^2^ + 2.4328*P*] where *P* = (*F*_o_^2^ + 2*F*_c_^2^)/3
2626 reflections	(Δ/σ)_max_ < 0.001
170 parameters	Δρ_max_ = 0.91 e Å^−^^3^
0 restraints	Δρ_min_ = −0.94 e Å^−^^3^

### Special details

**Table:**

Geometry. All e.s.d.'s (except the e.s.d. in the dihedral angle between two l.s. planes) are estimated using the full covariance matrix. The cell e.s.d.'s are taken into account individually in the estimation of e.s.d.'s in distances, angles and torsion angles; correlations between e.s.d.'s in cell parameters are only used when they are defined by crystal symmetry. An approximate (isotropic) treatment of cell e.s.d.'s is used for estimating e.s.d.'s involving l.s. planes.
Refinement. Refinement of *F*^2^ against ALL reflections. The weighted *R*-factor *wR* and goodness of fit *S* are based on *F*^2^, conventional *R*-factors *R* are based on *F*, with *F* set to zero for negative *F*^2^. The threshold expression of *F*^2^ > σ(*F*^2^) is used only for calculating *R*-factors(gt) *etc*. and is not relevant to the choice of reflections for refinement. *R*-factors based on *F*^2^ are statistically about twice as large as those based on *F*, and *R*- factors based on ALL data will be even larger.

### Fractional atomic coordinates and isotropic or equivalent isotropic displacement parameters (Å^2^)

**Table:**

	*x*	*y*	*z*	*U*_iso_*/*U*_eq_	Occ. (<1)
Pd1	0.0000	0.0000	0.0000	0.02988 (11)	
N1	0.1491 (3)	0.08511 (16)	0.1032 (4)	0.0310 (6)	
N2	−0.0576 (4)	0.14664 (17)	0.2356 (4)	0.0365 (7)	
H2N	−0.0894	0.1943	0.2789	0.055*	
N3	−0.1005 (3)	0.00901 (17)	0.2323 (4)	0.0300 (6)	
C1	0.3025 (4)	0.0847 (2)	0.0763 (5)	0.0377 (8)	
H1	0.3437	0.0388	0.0251	0.045*	
C2	0.4012 (5)	0.1469 (2)	0.1189 (6)	0.0445 (10)	
H2	0.5082	0.1445	0.0973	0.053*	
C3	0.3411 (5)	0.2138 (2)	0.1947 (6)	0.0456 (10)	
H3	0.4055	0.2590	0.2214	0.055*	
C4	0.1882 (5)	0.2140 (2)	0.2306 (5)	0.0397 (9)	
H4	0.1461	0.2588	0.2852	0.048*	
C5	0.0942 (4)	0.1478 (2)	0.1864 (5)	0.0339 (8)	
C6	−0.1315 (4)	0.0808 (2)	0.2959 (5)	0.0315 (7)	
C7	−0.2348 (4)	0.0899 (2)	0.4275 (5)	0.0410 (9)	
H7	−0.2600	0.1412	0.4681	0.049*	
C8	−0.2983 (5)	0.0241 (3)	0.4962 (5)	0.0453 (10)	
H8	−0.3703	0.0293	0.5832	0.054*	
C9	−0.2576 (5)	−0.0503 (2)	0.4390 (5)	0.0429 (9)	
H9	−0.2977	−0.0966	0.4897	0.052*	
C10	−0.1587 (4)	−0.0558 (2)	0.3084 (5)	0.0375 (8)	
H10	−0.1299	−0.1068	0.2697	0.045*	
N4	0.7615 (4)	0.32688 (19)	0.3476 (5)	0.0396 (7)	
O1	0.7104 (4)	0.39245 (17)	0.3829 (4)	0.0598 (9)	
O2	0.9002 (4)	0.3157 (2)	0.3252 (5)	0.0657 (10)	
O3A	0.683 (2)	0.2662 (8)	0.380 (8)	0.078 (7)	0.55 (10)
O3B	0.6702 (18)	0.278 (2)	0.282 (10)	0.073 (10)	0.45 (10)

### Atomic displacement parameters (Å^2^)

**Table:**

	*U*^11^	*U*^22^	*U*^33^	*U*^12^	*U*^13^	*U*^23^
Pd1	0.03068 (19)	0.02516 (18)	0.0353 (2)	−0.00072 (16)	0.01034 (15)	−0.00249 (17)
N1	0.0332 (15)	0.0272 (14)	0.0335 (16)	−0.0007 (12)	0.0078 (13)	−0.0013 (12)
N2	0.0405 (17)	0.0276 (15)	0.0430 (18)	0.0000 (13)	0.0123 (15)	−0.0075 (13)
N3	0.0306 (14)	0.0337 (16)	0.0263 (13)	−0.0011 (12)	0.0053 (11)	−0.0025 (12)
C1	0.0331 (19)	0.043 (2)	0.038 (2)	−0.0019 (16)	0.0103 (16)	0.0033 (17)
C2	0.035 (2)	0.052 (2)	0.047 (2)	−0.0079 (18)	0.0044 (18)	0.0068 (19)
C3	0.048 (2)	0.038 (2)	0.051 (3)	−0.0123 (18)	0.004 (2)	0.0012 (19)
C4	0.049 (2)	0.0308 (19)	0.040 (2)	−0.0059 (16)	0.0049 (18)	−0.0019 (16)
C5	0.0367 (19)	0.0319 (18)	0.0335 (19)	−0.0007 (15)	0.0065 (16)	0.0019 (15)
C6	0.0332 (18)	0.0342 (18)	0.0266 (17)	0.0027 (14)	0.0015 (15)	−0.0057 (15)
C7	0.035 (2)	0.049 (2)	0.039 (2)	−0.0002 (17)	0.0079 (17)	−0.0094 (18)
C8	0.037 (2)	0.065 (3)	0.035 (2)	−0.0025 (19)	0.0103 (17)	0.0022 (19)
C9	0.044 (2)	0.050 (2)	0.035 (2)	−0.0117 (19)	0.0055 (18)	0.0070 (18)
C10	0.043 (2)	0.0353 (19)	0.035 (2)	−0.0049 (16)	0.0053 (17)	0.0057 (16)
N4	0.0394 (18)	0.0362 (17)	0.0433 (19)	−0.0002 (14)	0.0051 (15)	−0.0024 (15)
O1	0.079 (2)	0.0378 (16)	0.066 (2)	0.0150 (15)	0.0226 (18)	0.0034 (15)
O2	0.0398 (17)	0.089 (3)	0.071 (2)	0.0008 (17)	0.0179 (16)	−0.0229 (19)
O3A	0.076 (6)	0.035 (4)	0.12 (2)	−0.016 (4)	0.006 (9)	0.008 (6)
O3B	0.051 (6)	0.048 (7)	0.12 (3)	−0.020 (5)	−0.006 (8)	−0.007 (11)

### Geometric parameters (Å, º)

**Table:**

Pd1—N1^i^	2.021 (3)	C3—H3	0.9500
Pd1—N1	2.021 (3)	C4—C5	1.397 (5)
Pd1—N3	2.030 (3)	C4—H4	0.9500
Pd1—N3^i^	2.030 (3)	C6—C7	1.404 (5)
N1—C5	1.340 (4)	C7—C8	1.363 (6)
N1—C1	1.353 (4)	C7—H7	0.9500
N2—C6	1.381 (4)	C8—C9	1.384 (6)
N2—C5	1.391 (4)	C8—H8	0.9500
N2—H2N	0.9200	C9—C10	1.366 (5)
N3—C6	1.341 (4)	C9—H9	0.9500
N3—C10	1.355 (4)	C10—H10	0.9500
C1—C2	1.364 (5)	N4—O3B	1.204 (15)
C1—H1	0.9500	N4—O1	1.231 (4)
C2—C3	1.390 (6)	N4—O2	1.234 (4)
C2—H2	0.9500	N4—O3A	1.264 (17)
C3—C4	1.367 (5)		
			
N1^i^—Pd1—N1	180.00 (19)	C3—C4—H4	120.2
N1^i^—Pd1—N3	94.06 (11)	C5—C4—H4	120.2
N1—Pd1—N3	85.94 (11)	N1—C5—N2	119.9 (3)
N1^i^—Pd1—N3^i^	85.94 (11)	N1—C5—C4	121.3 (3)
N1—Pd1—N3^i^	94.06 (11)	N2—C5—C4	118.7 (3)
N3—Pd1—N3^i^	180.0	N3—C6—N2	119.7 (3)
C5—N1—C1	118.0 (3)	N3—C6—C7	120.9 (3)
C5—N1—Pd1	120.0 (2)	N2—C6—C7	119.3 (3)
C1—N1—Pd1	121.7 (2)	C8—C7—C6	119.0 (4)
C6—N2—C5	125.1 (3)	C8—C7—H7	120.5
C6—N2—H2N	115.1	C6—C7—H7	120.5
C5—N2—H2N	113.8	C7—C8—C9	120.0 (4)
C6—N3—C10	119.0 (3)	C7—C8—H8	120.0
C6—N3—Pd1	119.5 (2)	C9—C8—H8	120.0
C10—N3—Pd1	120.7 (2)	C10—C9—C8	118.7 (4)
N1—C1—C2	123.4 (4)	C10—C9—H9	120.6
N1—C1—H1	118.3	C8—C9—H9	120.6
C2—C1—H1	118.3	N3—C10—C9	122.1 (4)
C1—C2—C3	118.3 (4)	N3—C10—H10	118.9
C1—C2—H2	120.9	C9—C10—H10	118.9
C3—C2—H2	120.9	O3B—N4—O1	118.3 (11)
C4—C3—C2	119.2 (4)	O3B—N4—O2	115.7 (10)
C4—C3—H3	120.4	O1—N4—O2	122.6 (4)
C2—C3—H3	120.4	O1—N4—O3A	118.7 (9)
C3—C4—C5	119.5 (4)	O2—N4—O3A	116.2 (8)
			
N3—Pd1—N1—C5	−43.5 (3)	C6—N2—C5—N1	36.1 (5)
N3^i^—Pd1—N1—C5	136.5 (3)	C6—N2—C5—C4	−141.9 (4)
N3—Pd1—N1—C1	142.8 (3)	C3—C4—C5—N1	−2.5 (6)
N3^i^—Pd1—N1—C1	−37.2 (3)	C3—C4—C5—N2	175.4 (4)
N1^i^—Pd1—N3—C6	−133.9 (3)	C10—N3—C6—N2	172.0 (3)
N1—Pd1—N3—C6	46.1 (3)	Pd1—N3—C6—N2	−18.3 (4)
N1^i^—Pd1—N3—C10	35.6 (3)	C10—N3—C6—C7	−6.0 (5)
N1—Pd1—N3—C10	−144.4 (3)	Pd1—N3—C6—C7	163.7 (3)
C5—N1—C1—C2	−4.4 (6)	C5—N2—C6—N3	−33.2 (5)
Pd1—N1—C1—C2	169.4 (3)	C5—N2—C6—C7	144.9 (4)
N1—C1—C2—C3	0.4 (6)	N3—C6—C7—C8	2.9 (6)
C1—C2—C3—C4	2.6 (6)	N2—C6—C7—C8	−175.1 (4)
C2—C3—C4—C5	−1.6 (6)	C6—C7—C8—C9	1.5 (6)
C1—N1—C5—N2	−172.5 (3)	C7—C8—C9—C10	−2.7 (6)
Pd1—N1—C5—N2	13.6 (5)	C6—N3—C10—C9	4.9 (5)
C1—N1—C5—C4	5.4 (5)	Pd1—N3—C10—C9	−164.7 (3)
Pd1—N1—C5—C4	−168.5 (3)	C8—C9—C10—N3	−0.5 (6)

Symmetry code: (i) −*x*, −*y*, −*z*.

### Hydrogen-bond geometry (Å, º)

**Table:**

*D*—H···*A*	*D*—H	H···*A*	*D*···*A*	*D*—H···*A*
N2—H2*N*···O2^ii^	0.92	2.08	2.964 (4)	160
N2—H2*N*···O3*A*^ii^	0.92	2.49	3.274 (17)	144
C2—H2···O1^iii^	0.95	2.57	3.409 (5)	148
C3—H3···O3*A*	0.95	2.55	3.23 (4)	129
C4—H4···O2^ii^	0.95	2.37	3.152 (5)	140
C7—H7···O3*A*^ii^	0.95	2.25	3.07 (2)	144
C10—H10···O2^iv^	0.95	2.53	3.334 (5)	142

Symmetry codes: (ii) *x*−1, *y*, *z*; (iii) *x*, −*y*+1/2, *z*−1/2; (iv) −*x*+1, *y*−1/2, −*z*+1/2.
